# Supplementary material for: Dual-Functional Polymeric Micelles Co-Loaded with Antineoplastic Drugs and Tyrosine Kinase Inhibitor for Combination Therapy in Colorectal Cancer
Source: Pharmaceutics. 2022 Mar 31;14(4):768. doi: 10.3390/pharmaceutics14040768 (PMC9030189; doi:10.3390/pharmaceutics14040768)
Supplement: Supplementary file 1 [file pharmaceutics-14-00768-s001.zip › pharmaceutics-1635149-supplementary.pdf]

pharmaceutics

MDPI

Supplementary Materials: Dual-Functional Polymeric Micelles Co-Loaded with Antineoplastic Drugs and Tyrosine Kinase Inhibitor for Combination Therapy in Colorectal Cancer

Ying-Hsia Shih, Cheng-Liang Peng, Ping-Fang Chiang and Ming-Jium Shieh

Table S1. Characterization of SN-38 micelles, Sunitinib micelles and SN-38/Sunitinib micelles by different drug/polymer ratio.

Abbreviations: D/P, Drug to polymer ratio. PDI, Polydispersity index.

Polymeric Micelles Formulations	D/P	Mean Size (nm)	PDI
SN-38 micelles	0.25/10	271.5	0.220
	0.5/10	268.1	0.213
	1/10	163.6	0.150
Sunitinib micelles	0.25/10	97.5	0.113
	0.5/10	99.2	0.129
	1/10	97.7	0.109
SN-38/Sunitinib micelles	0.25/1/10	89.2	0.106
	0.25/2/10	92.3	0.116
	0.5/1/10	88.8	0.142
	0.5/2/10	92.4	0.099
	1/1/10	112.8	0.134

Abbreviations: D/P, Drug to polymer ratio. PDI, Polydispersity index.

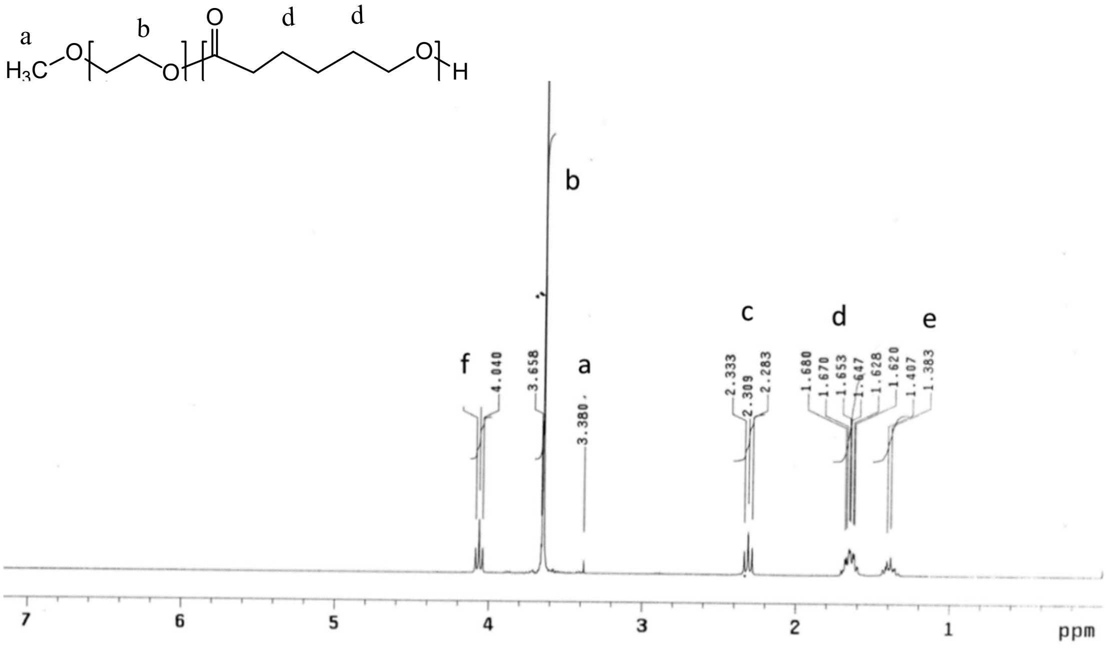

Figure S1. ^{1}H NMR spectra of mPEG-PCL polymer in $CDCl_3$.

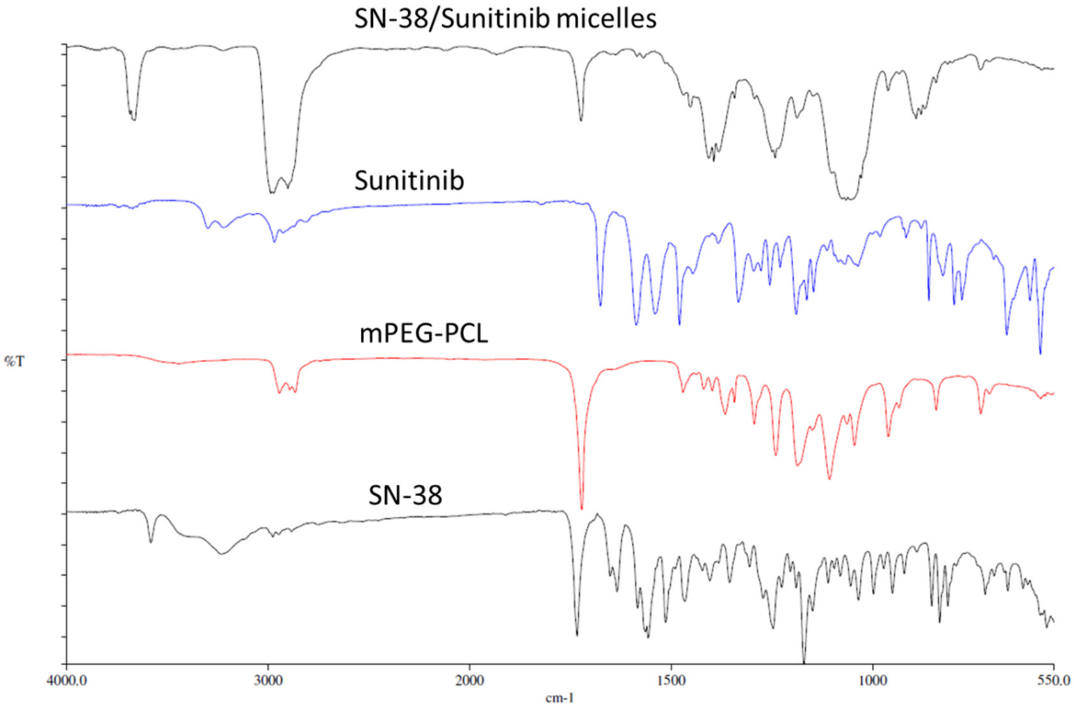

Figure S2. FTIR spectra of SN-38/Sunitinib micelles, Sunitinib, mPEG-PCL and SN-38 scanned within 550-4000 cm^{-1}.

Abbreviations: FTIR, Fourier-transform infrared spectroscopy. mPEG-PCL, methoxy poly-(ethylene glycol)-poly (ε-caprolactone).

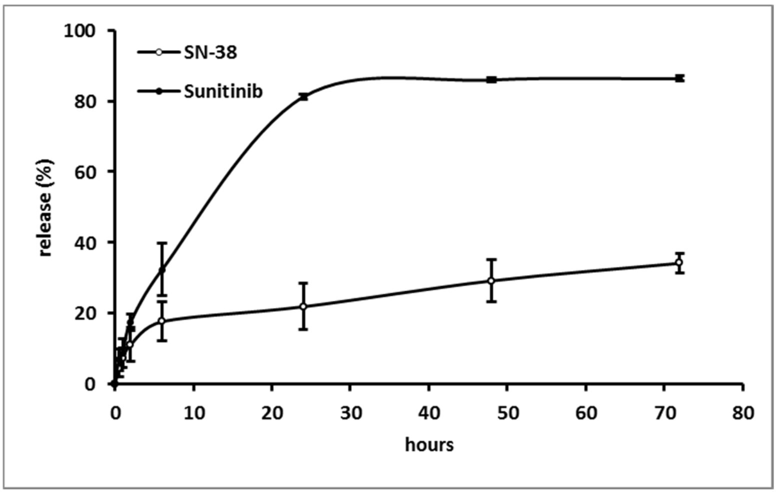

Figure S3. Release profile of synthesized SN-38/Sunitinib co-loading micelles. SN-38/Sunitinib micelles (D/P ratio at 0.25/2/10) samples which were measured every day for 3 days.

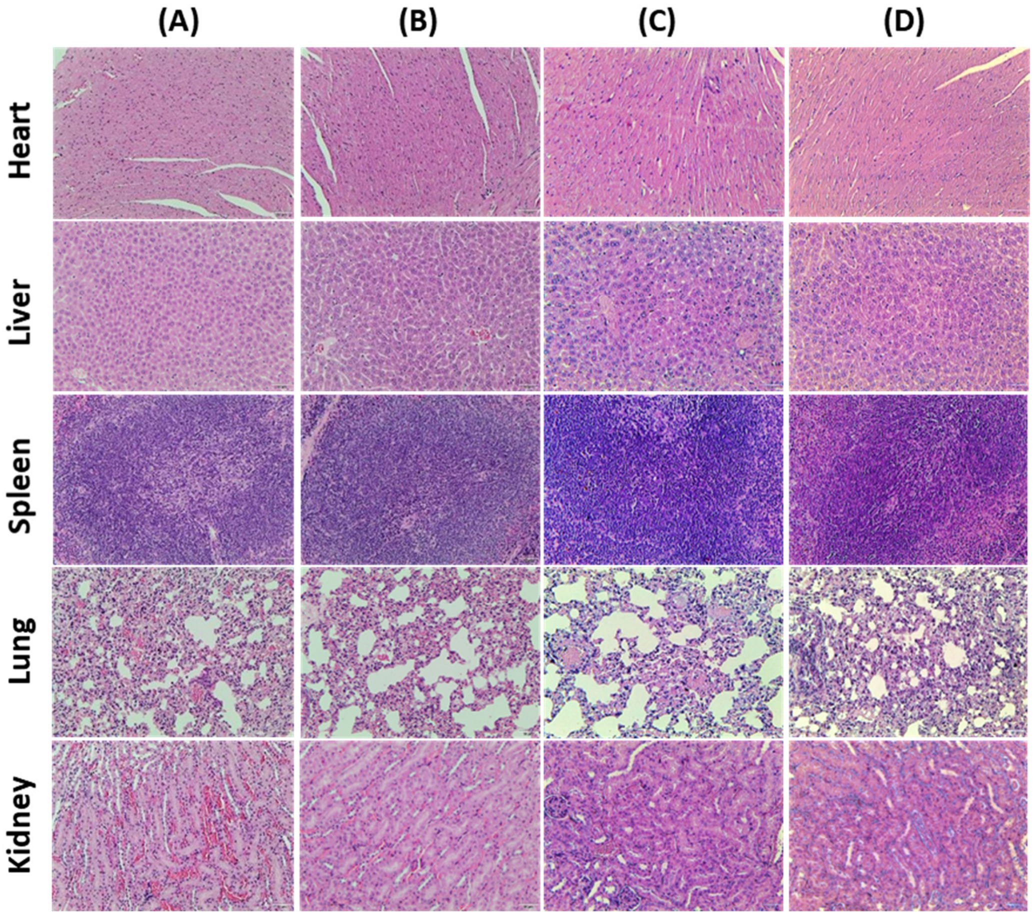

Figure S4. Histochemical staining of the main organs from mice at 28 days after treatment comprising control (A), SN-38 micelles (B), Sunitinib micelles (C), SN-38/Sunitinib micelles (D). Main tissues and HCT-116 tumor of mice sections were analyzed using hematoxylin and eosin (H&E, 200X magnification).
